# Pitfalls and Safeguards in Industry-Funded Research

**DOI:** 10.31486/toj.19.0093

**Published:** 2020

**Authors:** Joseph L. Breault, Emily Knafl

**Affiliations:** ^1^ Former Chair, Institutional Review Board, and Senior Physician, Department of Family Medicine, Ochsner Clinic Foundation, New Orleans, LA; ^2^ The University of Queensland Faculty of Medicine, Ochsner Clinical School, New Orleans, LA

**Keywords:** *Bias*, *conflict of interest*, *ethics*, *research*

## Abstract

**Background:** Physicians should follow ethical principles in their relationships with industry and be mindful that such relationships—if they are perceived as conflicts of interest—can undermine trust in the patient-physician relationship.

**Methods:** By identifying potential pitfalls and safeguards that can help prevent problems, this article focuses on ensuring that physician-industry relationships do not result in ethical transgressions or cause damage to doctor-patient relationships.

**Results:** Patient trust in physicians can be undermined by the perception that a physician-investigator is operating in the best interest of the research rather than the best interest of the patient. Payments from the pharmaceutical industry to physician-investigators are transparent because of the Sunshine Act, and patients can easily determine if their personal physicians have received money from industry. Research subsidies from industry should represent fair market value for the work performed. Postmarketing trials with the primary goal of increasing familiarity with a drug and prescribing rates should be avoided. Medical societies play an important role in establishing standards for professional conduct.

**Conclusion:** Ethically sound actions in physician relationships with industry should be guided by professional standards, medical society guidelines, and local institutional policies.

## INTRODUCTION

Because research is the foundation upon which medical advances are made, the driving forces in research funding deserve special scrutiny. The overwhelming majority of research funding comes from the government and industry, but the industry proportion is growing. The distribution in funding shifted from 46% originating from private industry sponsorship in 1994 to 59% in 2012.^1^ Industry sponsorship has continued to expand as government funding has persistently decreased.^1^

Patient trust in physicians has also declined.^2^ Historic polling shows that the percentage of patients reporting they had “great confidence in the leaders of the medical profession” declined from 73% in 1966 to 34% in 2012.^2^ The erosion of the public view of medicine is multifactorial and likely includes elements such as rising health costs, the opioid crisis, shortened clinic times, and shifting cultures. However, one factor that has been shown to influence patient opinion is their perception of physicians’ conflicts of interest, especially sponsorships from for-profit entities.^3^ An inverse relationship exists between how much funding a physician is perceived to have received from industry and how high a trust rating patients assign to him/her.^3^

Public media scrutiny has influenced this decline in trust. A 2012 article in *The Washington Post* told the story of the diabetes drug Avandia (rosiglitazone). Reporting in the *New England Journal of Medicine*, researchers heralded the drug as performing best among several medications but did not report data suggesting an increased risk of heart attacks.^4^ *The Washington Post* insinuated a strong correlation between the *New England Journal* article and the fact that much of the research funding came from GlaxoSmithKline. The drug was widely prescribed, and according to the *Washington Post*, a US Food and Drug Administration (FDA) scientist estimated that the drug was associated with 83,000 heart attacks and deaths before it was removed from the US market.^4^ Emails uncovered and released by the US Senate showed that GlaxoSmithKline suppressed data when sharing information with academic researchers and deliberately avoided further clinical trials that may have revealed detrimental side effects.^4^

A 2006-2007 article series aired by National Public Radio (NPR) focused on the painkiller Vioxx (rofecoxib) and its correlation to heart attacks and strokes.^5,6^ NPR reported that the studies were conducted on patient populations too small to sufficiently assess the risks potentially associated with the drug despite early evidence that the cardiac risk of the medication was twice as high in the Vioxx group vs those receiving naproxen. Once the connection between Vioxx and heart attacks became public, Merck withdrew the drug from the market in 2004 and agreed to a $4.85 billion lawsuit settlement.^5,6^

Because of these reports and others, a 2016 study by Klein et al showed that 61% of patients who had never been in a clinical study reported they would be less likely to trust a physician who received industry payments for the study.^7^

As Tiereny et al explained, “The goal of federal research funding is to generate new knowledge that will enhance health and health care. The goal of research funding by for-profit companies is maximizing income to shareholders.”^8^ These goals are not necessarily mutually exclusive, and in many instances, they can act in harmony to serve patients. When examining these delicate dynamics, recognizing that industry has a role to play in research that is not necessarily at odds with the interests of patients is important. For example, the National Institutes of Health (NIH) has partnered with the FDA and several industry partners in an alliance called the Accelerating Medicines Partnership (AMP) with the aim of increasing the availability of diagnostics and therapies to patients.^9^ The former US Health and Human Services (HHS) Secretary Kathleen Sebelius stated, “To accelerate our nation's therapeutic development process, it is essential that we forge strong, innovative, and strategic partnerships across government, academia, and industry.”^10^ Increasing the potential for discovery of therapies and expanding medical knowledge to benefit patients are the ultimate goals, and industry contributes to them.

By identifying potential pitfalls and safeguards that can help prevent such problems, this article focuses on ways to ensure that physician-industry relationships do not result in ethical transgressions or cause damage to doctor-patient relationships.

## PITFALLS ASSOCIATED WITH INDUSTRY-FUNDED RESEARCH

### Publication and Data Bias

One concerning trend in industry-funded research is the higher likelihood of positive data results.^11^ In a 2003 systematic review, Lexchin et al suggest at least four possible explanations for favorable results in industry-sponsored research: (1) companies may selectively fund trials on drugs that they consider to be superior to the competition, (2) positive results could be the consequence of poor quality research, (3) the doses of the study and comparator drugs may not be equivalent, and (4) research sponsored by industry is less likely to be published than research with other sources of funding.^11^ Rochon et al reported that in most cases of nonequivalent doses they examined, the study drug was given at the higher dose, potentially biasing the results in favor of the manufacturer's product.^12^ McCarthy reported on a company's attempts to block the publication of an article showing that its human immunodeficiency-1 vaccine was no better than placebo.^13^

### Postmarketing Research

Some postmarketing trials are initiated with the primary intent to create familiarity with a particular product rather than to determine any scientific outcome or stated objective—marketing wearing the mask of science. Ross et al reviewed litigation against Merck and found evidence that a postmarketing trial was initiated to promote the prescription of Vioxx.^14^ The purpose of the trial, in the words of Merck's marketing executives, was “to provide product trial among a key physician group to accelerate uptake of Vioxx as the second entrant in a highly competitive new class and [the trial] was designed and executed in the spirit of the Merck marketing principles.”^14^

Phase IV clinical trials can expand safety and efficacy data important to FDA labeling updates, especially when the FDA requests such trials after recognizing the need for them. However, non–FDA-mandated phase IV studies that appear to have little scientific benefit can be mechanisms for marketing to doctors. Evidence shows that participation in trials shapes physicians’ prescribing habits by increasing their familiarity with the drug and subconsciously creating a personal investment in the product's success.^15-17^

### Funding Bias

Physician participation in pharmaceutical company–funded clinical trials is essential to medical progress. These physician researchers can become experts in the study drugs or devices and may be invited to join speakers’ bureaus, advisory committees, and boards of directors—and be compensated for doing so. Interactions that involve money transfers from pharmaceutical companies to research physicians can be viewed as potential conflicts of interest.

### Excessive Compensation

The HHS Office of Inspector General (OIG) compliance guidance for research funding states that contracts between sponsors and investigators/institutions conducting research on their behalf “…should be structured to fit in the personal services safe harbor whenever possible. Payments for research services should be fair market value for legitimate, reasonable, and necessary services. Post-marketing research activities should be especially scrutinized to ensure that they are legitimate and not simply a pretext to generate prescriptions of a drug.”^18^

The OIG personal services safe harbor is codified at 42 CFR §1001.952(d) and includes 7 standards. Standard 5 reads as follows:

The aggregate compensation paid to the agent over the term of the agreement is set in advance, is consistent with fair market value in arms-length transactions and is not determined in a manner that takes into account the volume or value of any referrals or business otherwise generated between the parties for which payment may be made in whole or in part under Medicare, Medicaid or other Federal health care programs.^19^

### Double Billing

Double billing is billing twice for services or procedures in a research trial: once to the sponsor and again to the research subject or the subject's insurance company. When the insurance company is Medicare/Medicaid, such double billing means fraudulently billing the federal government, an offence that falls under the jurisdiction of the False Claims Act (FCA). Even if the double billing results from inadequate controls without an intent to commit fraud, the civil FCA may still allow “fines up to three times the programs’ loss plus $11,000 per claim filed. Under the civil FCA, each instance of an item or service billed to Medicare or Medicaid counts as a claim.”^20^ In 2013, Emory University agreed to pay a $1.5 million settlement after a double-billing investigation. A Department of Justice (DOJ) announcement stated, “the United States and the State of Georgia alleged that Emory University billed Medicare and Medicaid for services the clinical trial sponsor agreed to pay (and, in some cases, actually did pay, thereby resulting in Emory's being paid twice for the same service).”^21^

The criminal FCA may add imprisonment to the fines when the DOJ deems that a criminal case is warranted. The following example is from a joint OIG-DOJ 2016 report:

…in April 2016, one of our [pharmaceutical fraud program] investigations opened in FY2014 resulted with a clinical trial coordinator being arrested and charged for falsifying documents within multiple clinical trials he was controlling. The investigator was forging the primary investigator's signature and “back dating” the forms he submitted to drug sponsors in order to receive payments. It is estimated that the investigator profited nearly $500,000 from his actions.^22^

Even without double billing, if a research procedure billed to Medicare/Medicaid is later judged to be a not-covered service under the programs, the billing is fraudulent and subject to FCA penalties. After discovering that Medicare had been billed for nonreimbursable services in oncology clinical trials, Rush University Medical Center in Chicago self-reported the billing errors and agreed to pay $1 million to Medicare/Medicaid to settle the overpayment.^23^

### Insider Trading

Clinical trials personnel receive nonpublic reports from sponsors. These reports include information about serious adverse events and deaths that may result in the clinical trial being suspended. The reports may include midstudy analyses of nonefficacy that later result in a public cancellation of the trial. Such events can significantly affect the sponsor's stock price. If physicians and clinical trials personnel hold stock in the company, they may be tempted to sell the stock before the bad information becomes public to avoid a financial loss. This practice is illegal. Physician-investigators have been convicted of insider trading because of stock trades initiated after they received nonpublic reports of adverse events.^24^

### Physician Coercion

A physician may hold more influence over patients than he or she realizes. The physician-investigators participating in an industry-sponsored trial are responsible for overseeing recruitment and ensuring that an adequate number of patients are enrolled. Often, the principal source of recruitment is the physician's own patients, and this situation may pose the ethical concern of unintended coercion. Physicians may unintentionally coerce their patients into participating in a research study through the unbalanced authority and power in the relationship. Patients may enroll against their best judgment out of respect for their doctor and their level of trust in the relationship.

The HHS OIG discussed the potential problem with patient participation in physician-investigator research studies in the report *Recruiting Human Subjects: Pressures in Industry-Sponsored Clinical Research*:

We heard significant concerns that the dual role of physician-investigators might infringe upon this voluntariness, concerns worthy of particular attention as we found that investigators often enroll many of their own patients into their trials. Patients may be reluctant to contradict their doctor's wishes by refusing participation in a trial, or may agree to participate because they trust and respect their physician, who they believe is looking out for their best interests. As one coordinator we spoke with said, “patients see their doctor as God.” Another investigator recognized the trust patients hold in their doctors. He told us that he was reluctant to even mention to his patients a trial that involved withdrawing their asthma medications. He was afraid they would agree to participate because he asked them, despite the fact that their current medications were stabilizing their asthma. A Presidential advisory commission went so far as to state that the patients of physician-investigators should be considered a vulnerable population.^25^

## SAFEGUARDS

### Increased Government Funding

One solution to balance the increasing influence of industry-funded research is increased federal research funding. However, in recent decades, federal funding has been outpaced by inflation.^8^ Researchers can continue to request increased funding from their elected representatives.

### Increased Transparency

One source of transparency is ProPublica. As of March 2020, the ProPublica Dollars for Docs database included details about $12 billion in payments to doctors and teaching hospitals that have been disclosed by pharmaceutical companies and their subsidiaries since 2009.^26^

These data are available because of the Sunshine Act, a provision of the Affordable Care Act, that requires individual physicians, healthcare systems, and industry to disclose their financial relationships.^27^ A 2014 article in *Newsweek* explained that patients can use this information to identify their doctors’ potential financial conflicts of interest:

Most people know less about their doctor than they do about their local barista or bartender. The world of medicine—from the pediatrician checking your child's breathing to the specialist researcher toiling away at a university lab—is guardedly private. But that's about to change. Starting September 30, Jane and Joe Citizen will be able to search a government-run website and see all the consulting fees, stock options and trips to Key West given by pharmaceutical companies to primary care physicians, ob/gyns, dermatologists and other doctors. Referred to as the Sunshine Act, this transparency clause in the Affordable Care Act allows the Centers for Medicare & Medicaid Services to publicly post all payments and other valuables given by Big Pharma to physicians and teaching hospitals.^28^

Another example of safeguarding via transparency is the NIH-AMP alliance that requires all data and analyses to be publicly accessible to the biomedical community.^9,10^

### Medical Societies’ Ethics Guidelines

In *Clinical Ethics: A Practical Approach to Ethical Decisions in Clinical Medicine*, Jonsen et al define fiduciaries as those who have “specialized expertise and are held to high standards of honesty, confidentiality, and loyalty. Above all, fiduciaries must avoid financial conflicts of interest that could prejudice their clients’ interests.”^29^

Physicians are fiduciaries for their patients’ best interests, and professional societies such as the American College of Physicians (ACP) and the American Medical Association (AMA) have developed guidelines for physicians to follow in their medical practices (Table).^30,31^ The AMA Code of Medical Ethics Opinion 11.2.2 states, “The primary objective of the medical profession is to render service to humanity; reward or financial gain is a subordinate consideration. Under no circumstances may physicians place their own financial interests above the welfare of their patients.”^32^ The AMA states that trust is central to the relationship: “The relationship between a patient and a physician is based on trust, which gives rise to physicians’ ethical responsibility to place patients’ welfare above the physician's own self-interest or obligations to others.”^33^

Similarly, the ACP Ethics Manual is intended to guide physicians in making ethical decisions.^30^

**Table.** Medical Societies’ Current Ethical Standards: Research

American College of Physicians – Sponsored Research	All scientists are bound by the obligations of honesty and integrity in their research. However, in the high-stakes arena of the health care industry, industry-sponsored research is at greater risk for conflicts of interest. Scientists have a responsibility to protect human subjects, implement applicable research standards and privacy and confidentiality protections, register trials, interpret results objectively, submit their work for peer review, and disclose all conflicts of interest. With industry-sponsored research, scientists have the further obligations of ensuring that the entire data set is available and analyzed independently of the sponsor.^30^
American Medical Association – Conflicts of Interest in Research Code of Medical Ethics Opinion 7.1.4	Increasing numbers of physicians, both within and outside academic health centers, are becoming involved in partnerships with industry to conduct biomedical and health research. As they do so, physicians must be mindful of the conflicts such engagement poses to the integrity of the research and the welfare of human participants. In addition to financial conflicts of interest created by incentives to conduct trials and recruit subjects, physicians must be sensitive to the differing roles of clinician and investigator, which may require them to balance dual commitments to participants and science. This conflict of commitment is particularly acute when a physician-investigator has treated or continues to treat a patient who is eligible to enroll as a participant in a clinical trial the physician is conducting.
	Minimizing and mitigating conflicts of interest in clinical research is imperative if the medical community is to justify and maintain trust in the medical research community.
	Physicians who engage in research should:
	(a) Decline financial compensation that awards in excess of the physician's research efforts and does not reflect fair market value. Physicians should not accept payment solely for referring patients to research studies.
	(b) Ensure that the research protocol includes provision for funding participants’ medical care in the event of complications associated with the research. A physician should not double bill a third-party payer for additional expenses related to conducting the trial if he or she has already received funds from a sponsor for those expenses.
	(c) As part of the informed consent process, disclose to prospective participants the nature and source of funding and financial incentives offered to the investigators. This disclosure should be included in any written consent materials.
	(d) Avoid engaging in any research where there is an understanding that limitations can be placed on the presentation or publication of results by the research sponsor.
	(e) Refrain from knowingly participating in a financial relationship with a commercial entity with whom they have a research relationship until the research relationship ends and the research results have been published or otherwise disseminated to the public.
	(f) Disclose material ties to companies whose products they are investigating or other ties that create real or perceived conflicts of interest to:
	1. Institutions where the research will be carried out
	2. Organizations that are funding the research
	3. Any journal or publication where the research results are being submitted
	(g) Physicians who have leadership roles in institutions that conduct biomedical and health research as well as the entities that fund research with human participants should promote the development of guidelines on conflicts of interest that clarify physician-investigators responsibilities.^31^

### Institutional Review Committees

Healthcare institutions have multiple committees tasked with safeguarding proper procedures and ethics. These committees are another protective mechanism that help prevent potential conflicts of interest in research and include the following:

- The contracts and grants office checking that financial contracts are appropriate, including fair market value for investigator reimbursement
- The institutional review board (IRB) checking for adequate protection for human subjects
- The research conflict of interest committee (RCOIC) finding remedies to real or perceived financial conflicts of interest
- Hospital purchasing committees excluding physicians and others with real or perceived conflicts of interest from decisions about products being purchased

Physician-investigators in private practice might lack the safeguards of these institutional committees, except for an external IRB. These safeguard gaps can be challenging to navigate ethically.

The IRB may be able to help prevent phase IV clinical trials that have no clear scientific benefit and have not been requested by FDA. Sox and Rennie provide suggestions for how an IRB panel can help prevent these trials.^34^ One approach is to ask directly if a study is in fact a marketing trial. Another approach is to look for potential red flags: trials aimed to address questions already adequately addressed by other studies, lack of control groups, disproportionately large enrollment projections, and short timeframes for studying chronic disease. Sox and Rennie acknowledge that none of these findings is particularly specific, but a combination of them should prompt further questioning.

Physician influence over patients can be mitigated on an institutional level as well. One practice that may help to prevent coercion is to designate someone other than the attending physician to recruit patients for a research study.

Regarding financial conflicts, the accreditation body for IRBs—the Association for the Accreditation of Human Research Protection Programs—has a standard that addresses financial conflicts of interest for researchers and research staff:

Processes to define financial conflict of interest are generally dictated by laws, regulations, and codes, and generally vary in terms of what financial interests must be disclosed and when a financial interest is considered a financial conflict of interest. Organizations should have policies and procedures to manage or eliminate the financial conflicts of interest of Researchers and Research Staff that meet the laws, regulations, and codes to which they are bound.^35^

Thus, IRB members ask investigators about their financial interests in the industry sponsor (stock, patents/royalties, income from speakers’ bureaus or advisory committees). When a financial interest is present, the RCOIC reviews it to decide if it is a real or perceived conflict of interest, and, if it is real, how to manage or eliminate it. After the investigator agrees to the RCOIC management plan, the process moves to the IRB. One aspect of IRB approval is determining if the conflict of interest management plan appropriately protects research participants. If the IRB determines that the plan is inadequate, additional requirements or even the elimination of the investigator from the study may be required. The process varies from one intuition to another.

IRBs are federally mandated, but local IRBs are extensions of the local institution's authority. If an institution allows a central or offsite IRB to oversee the study, the institution still retains its role in managing the site's human research protection program. Commonly, the institution will maintain the RCOIC function to meet regulatory requirements that its investigators are not compromised in their research efforts by real or perceived conflicts of interest. The RCOIC has access to a database of annual conflict-of-interest filings from staff physicians and from the Sunshine Act. These data are checked against conflict of interest answers on the IRB application.

The PhRMA (Pharmaceutical Research and Manufacturers of America) Principles on Conduct of Clinical Trials states, “Clinical investigators or their immediate family should not have a direct ownership interest in the specific pharmaceutical product being studied.”^36^ The American College of Obstetricians and Gynecologists (ACOG) policy on professional relationships with industry states: “Once a clinical investigator becomes involved in a research project for a company or knows that he or she might become involved, the investigator, as an individual, cannot ethically buy or sell the company's stock until the results of the research are published or otherwise disseminated to the public and the involvement ends.”^37^ This ACOG opinion reflects a 2010 version of the AMA ethics opinion^38^ that has since been revised to that shown in the Table.

### Fines and Penalties

Adequate legislation and enforcement for transgressions committed by individuals, companies, and institutions in the conduct of research are necessary.^5,6^ Adequate enforcement requires pathways for reporting and oversight to identify issues. Faunce et al went so far as to suggest the need for an independent statutory entity with the power to enact fines and criminal penalties and to initiate litigation in civil court.^39^

### Fair Market Value/Billing Compliance

Research subsidies from pharmaceutical companies should never exceed fair market value for the work performed. Nothing that is billed to the sponsor or that the contract states can be billed to the sponsor should be billed to research participants or their insurers. Nothing should be billed to Medicare/Medicaid unless a Medicare coverage analysis concludes that the programs allow the charges. Institutions can guard against inappropriate research subsidies by research administration review of the clinical trial budget and approving or adjusting the budget based on written standard operating procedures. Institutions can guard against FCA claims by ensuring robust coordination between research and billing offices and a strong Medicare coverage analysis for each clinical trial before the study begins. A separate compliance group should monitor adequate controls by ensuring that procedures are written and by conducing appropriate audits.

## CONCLUSION

Biomedical advances cannot be made by pharmaceutical companies alone or by academia alone. To make the greatest progress in medical care, uniting resources, knowledge, and brilliant minds across both industry and academia is necessary. Ensuring the integrity of research and preserving trust in the medical profession require careful safeguarding from pitfalls. Much of this safeguarding can be accomplished by following institutional policies, working with institutional committees, and following the research ethics guidelines of medical societies and the IRB.

## Notes

This article meets the Accreditation Council for Graduate Medical Education and the American Board of Medical Specialties Maintenance of Certification competencies for Patient Care, Medical Knowledge, and Systems-Based Practice.
